# Study on the Sleep-Improvement Effects of *Hemerocallis citrina* Baroni in *Drosophila melanogaster* and Targeted Screening to Identify Its Active Components and Mechanism

**DOI:** 10.3390/foods10040883

**Published:** 2021-04-17

**Authors:** Yuxuan Liang, Riming Huang, Yongchun Chen, Jing Zhong, Jie Deng, Ziyi Wang, Zhuojun Wu, Meiying Li, Hong Wang, Yuanming Sun

**Affiliations:** College of Food Science, South China Agricultural University, Guangzhou 510642, China; 20181145005@stu.scau.edu.cn (Y.L.); huangriming@scau.edu.cn (R.H.); 20172123002@stu.scau.edu.cn (Y.C.); zhongjinghh@gmail.com (J.Z.); dengjie@stu.scau.edu.cn (J.D.); wangziyi@stu.scau.edu.cn (Z.W.); wzj2017@stu.scau.edu.cn (Z.W.); lmy1982@scau.edu.cn (M.L.); gzwhongd@scau.edu.cn (H.W.)

**Keywords:** *Hemerocallis citrina* Baroni, liquid chromatography coupled with mass spectrometry sleep, *Drosophila melanogaster*, network pharmacology, mRNA expression

## Abstract

*Hemerocallis citrina* Baroni (HC) is an edible plant in Asia, and it has been traditionally used for sleep-improvement. However, the bioactive components and mechanism of HC in sleep-improvement are still unclear. In this study, the sleep-improvement effect of HC hydroalcoholic extract was investigated based on a caffeine-induced insomnia model in *Drosophila melanogaster* (*D. melanogaster*), and the ultrahigh-performance liquid chromatography coupled with electrospray ionization quadrupole Orbitrap high-resolution mass spectrometry (UHPLC-ESI-Orbitrap-MS) and network pharmacology strategy were further combined to screen systematically the active constituents and mechanism of HC in sleep-improvement. The results suggested HC effectively regulated the number of nighttime activities and total sleep time of *D. melanogaster* in a dose-dependent manner and positively regulated the sleep bouts and sleep duration of *D. melanogaster*. The target screening suggested that quercetin, luteolin, kaempferol, caffeic acid, and nicotinic acid were the main bioactive components of HC in sleep-improvements. Moreover, the core targets (Akt1, Cat, Ple, and Sod) affected by HC were verified by the expression of the mRNA of *D. melanogaster*. In summary, this study showed that HC could effectively regulate the sleep of *D. melanogaster* and further clarifies the multi-component and multi-target features of HC in sleep-improvement, which provides a new insight for the research and utilization of HC.

## 1. Introduction

Sleep is a complex physiological process influenced by many factors [1]. The sleep disorders of the population keep increasing in modern society because of the changes in lifestyle and environment [2]. Emerging evidence indicates that inadequate sleep can have many negative effects on health, such as obesity, heart disease, anxiety, and depression [3,4,5,6,7]. Epidemiological studies have shown that the prevalence of sleep disorders ranges from 3.9% to 22.0% in the world population [8,9,10,11]. Drug therapy is still the main method of sleep-improvement; however, more and more studies have paid attention to dietary supplements in avoiding dependence, addiction, and other side-effects induced by drugs [2,12,13,14,15].

Similar to mammalian sleep, circadian rhythms of *D. melanogaster* have been quantified as a sleep/wake activity and neuromodulation of sleep behavior [16], and the locomotor activity of *D. melanogaster* is often used to measure sleep/wake activity [17]. At present, the spontaneous locomotor activity of *D. melanogaster* is quantified using a PC-based locomotor activity monitoring system, which records the locomotor activity of individual *D. melanogaster* based on the interruption of an infrared beam. It is able to monitor the locomotor behavior of *D. melanogaster* over a longer period of time under standardized living conditions, as well as to measure the sleep parameters of *D. melanogaster* [18]. The previous studies have defined the duration of sleep, the number of sleep cycles, and the average duration of each sleep in Drosophila [13,19,20]. Therefore, models of *D. melanogaster* have been valuable in the discovery of novel sleep regulators [17].

*Hemerocallis citrina* Baroni (HC), as an edible plant, is widely grown in Asia with high economic value due to its good taste and health-promoting properties, and it has a long history of being used to ameliorate sleep-related disorders [21,22,23]. However, the unclear material basis and molecular mechanisms of HC in sleep-improvement restrict its further development and application. Phytochemical analysis revealed that HC contains many bioactive components such as flavonols, anthraquinones, and polyphenols [24]. Furthermore, previous studies have shown that HC possesses anti-depression and anti-inflammatory effects [23,25,26,27]. Among them, Du et al. reported that the main compounds responsible for its antidepressant activity are flavonoids (especially rutin and hesperidin) [22,28]. Furthermore, a few studies have indicated that some single compounds such as rutin, hyperoside, and caffeic acid might possess the potential function of improving sleep [29,30,31,32,33], though these studies mainly focused on the bioactivity of a single compound. However, food provides a characteristic combination of multiple nutrients in one group that work together to play an essential role. Therefore, we speculate that there are multiple compounds in HC that play a role in sleep-improvement.

With the development of pharmacology, bioinformatics, and network science, Hopkins proposed the concept of “network pharmacology” in 2008 [34], which provides novel insights to reveal the coordinated interaction among multi-components, multi-targets, and multi-pathways, and to deepen the understanding of complex diseases at the system level [35,36]. So, in this study, the sleep-improvement effect of HC was firstly investigated based on the caffeine-induced insomnia model in *D. melanogaster*. Then, high-resolution UHPLC-ESI-Orbitrap-MS was used to analyze the composition of HC. On this basis, the network pharmacology strategy was used to screen bioactive compounds and identify the mechanism of HC in sleep-improvement. Moreover, the transcription levels of core targets of the network were further verified.

## 2. Materials and Methods

### 2.1. Materials

Commercial *Hemerocallis citrina* Baroni (flower buds) was obtained from Qidong county, Hunan province. Acetonitrile hypergrade for liquid chromatography coupled with mass spectrometry (LC-MS) LiChrosolv^®^ was bought from Merck KgaA (Darmstadt, Germany), and methanol hypergrade was purchased from SIMARK (Tianjin, China). Ultrapure water was prepared using the Unique-R10 system from Research Water Purification Technology Co. Ltd. (Xiamen, China).

### 2.2. Preparation of HC Extract

Two hundred grams of HC was ground to powder and extracted three times in 2000 mL of 60% ethanol under a temperature of 25 °C, each time for 24 h. The combined ethanolic extract was centrifuged at 12,000 rpm for 15 min, and then the supernatant was concentrated under reduced pressure on a rotary evaporator (45 °C, 60 hpa). Part of the concentrate was lyophilized for 72 h to obtain lyophilized powder and stored at −20 °C. Additionally, part of the concentrate was filtered by a 0.22 μm filter membrane and then stored at −4 °C until UPLC-orbitrap-MS analysis.

### 2.3. Drosophila Stocks

Wild-type *D. melanogaster* (Canton-S strain) were purchased from the Core Facility of Drosophila Resources and Technology, Shanghai Institute of Biochemistry and Cell Biology, Chinese Academy of Sciences. The flies were raised in standard medium (5% sucrose, 8% cornmeal, 4% dried yeast, 1% agar, and 0.5% propionic acid) at 25 °C in 12:12 h light: dark cycles. Male flies (3-day-old) were collected under CO_2_ anesthesia for formal experiment.

### 2.4. Measurement of Locomotor Activity

The food used in the measurement of locomotor activity was made up of 1% agar and 5% sucrose. Agar-sucrose food containing 0.1% caffeine was made. On this basis, 0.625%, 1.250%, and 2.500% of HC extract were add to the agar-sucrose food containing 0.1% caffeine, respectively. Each group contained 19 fruit flies. *D. melanogaster* were individually transferred in separate plastic tubes and were monitored through the Drosophila Activity Monitoring System (DAM; TriKinetics, Waltham, MA, USA). Monitoring was performed for 3 days under conditions of constant darkness at 25 °C and 60% relative humidity, and locomotor activity was taken every minute. The sleep of *D. melanogaster* was defined as no locomotor activity observed within 5 min [37], and the number of sleep bouts and sleep duration was counted by excel [13,38].

### 2.5. UHPLC-Orbitrap-MS Analysis

A Q Exactive-Orbitrap Mass Spectrometer (Thermo Fisher Scientific, Bremen, Germany), equipped with an electrospray ionization (ESI) source working in a positive model and a negative model was used for accurate mass measurements. Liquid Chromatography analysis was performed using a Dionex UltiMate 3000 (Thermo Fisher Scientific, Bremen, Germany) equipped with a quaternary pump and a thermostated autosampler. Chromatographic separation was accomplished with an Agilent InfinityLab Poroshell 120, 4.6 mm × 150 mm, 2.7 µm column were used (Agilent Infinity Lab, Wilmington, DE, USA). The detailed operation was performed according to the previous method with slight modifications, and it is presented in the Supplementary Material [39].

### 2.6. Profiling of HC Constituents

Compounds of HC were monitored using TraceFinder™ software v3.4 (Thermo Fisher Scientific, Waltham, MA, USA), loaded with the Orbitrap traditional Chinese medicine library (OTCML) database to process the UHPLC-MS data [40]. The OTCML database refers to the Chinese medicinal materials included in the Chinese pharmacopoeia (2015 edition), and it contains the mass spectrometry data and chromatographic retention time information of more than 1200 kinds of traditional Chinese medicine compounds and more than 7000 high-quality high-resolution secondary mass spectrometry data [41]. A threshold signal of 1.0 × 10^6^ and an accurate mass measurement error lower than 5 ppm was established in the TraceFinder™ software to consider a positive match in the analyzed HC samples. In addition, compound confirmation was only granted if all the mentioned confirmation criteria (M/Z, isotopic pattern, and fragment ion) were accomplished after raw data processing with the TraceFinder™ screening software.

### 2.7. Collection of Candidate Targets

For the targets of HC identified compounds, the databases of Traditional Chinese Medicine System Pharmacology (TCMSP) [42], Swiss Target Prediction [43], SymMap [44], and PubChem [45] were adopted to screen the candidate targets. For targets of sleep-related indication, the candidate targets were collected from the database of GeneCards (version 4.14) [46], DrugBank (version 5.1.6, released 22 April 2020) [47], and DisGeNET (version 7.0) [48,49,50]. The score of the gene symbol for the GeneCards database was set at higher than 5.0, and the score of the gene symbol for the DisGeNET database was set at higher than 0.1. For the DrugBank database, keywords for sleep-related indications were “insomnia”, “sleep disorders and disturbance”, “rapid eye movement sleep disorder”, “non-24-h sleep-wake disorder”, “disturbed sleep nightmares”, “sleep initiation disorders”, “shift-work related sleep disturb”, and “difficulty sleeping”.

### 2.8. Functional Enrichment Analysis and Network Construction

Functional enrichment analysis of Kyoto Encyclopedia of Genes and Genomes (KEGG) pathway and Gene Ontology (GO) terms in biological process (BP), molecular function (MF), and cellular components (CC) from overlapped targets were performed by using STRING bioinformatics resources and the KEGG database [51]. To uncover the complex interaction among targets, compounds, and sleep-related pathway, a C-T-P network was constructed by using Cytoscape (version 3.6) [52]. In addition, the protein-protein interaction (PPI) network of *D. melanogaster* was constructed by intersection targets, and three topological indicators (Degree, Betweenness centralityand Closeness centrality) of the network were further analyzed by the Network Analyzer [53,54].

### 2.9. mRNA Expression

The *D. melanogaster* were fed with the corresponding diets (normal, caffeine, caffeine + 2.5% HC) in constant darkness for 3 days (*n* = 50). Total RNA was extracted from the heads of *D. melanogaster* using Trizol (GBCBIO Technologies, Guangzhou, China), according to the manufacture’s protocol. A reverse transcription reagent kit (TaKaRa, Dalian, China) was then used to obtain cDNA, and the mRNA expression levels were determined by RT-PCR using TB Green Premix Ex TaqII (TaKaRa, Dalian, China). The measured genes included Akt1, Sod, Cat, and Ple, and the Rp49 was used for the endogenous control [55,56]. The primer sequences have been displayed in Table S1. The relative expression levels were calculated by the ΔΔCt method.

### 2.10. Statistical Analysis

SPSS 20.0 (SPSS Inc., Chicago, IL, USA) was used for statistical analysis of the data. The Kolmogorov–Smirnov test was used to test the normality of data. If the data then fit a normal distribution, one-way analysis of variance was used to analyze statistically significant differences between groups. Otherwise, non-parametric tests (the Kruskal-Wallis test) were used. The data of locomotor activity are presented as mean ± standard error of the mean (SEM) for each group. When the variances are assumed to be consistent, the least significant difference (LSD) was used to analyze the differences between groups. When the variances were not assumed to be consistent, Tamhane’s T2 was used to analyze the differences between groups. It was considered statistically significant when *p* < 0.05. The statistical procedures of mRNA expression were consistent with the statistical procedures of measurement of locomotor activity (the Shapiro–Wilk normality test was used to test the normality of data). The data of mRNA expression are presented as mean ± standard deviation (SD) for each group.

## 3. Results and Discussion

### 3.1. Effect of HC on Locomotor Activity of D. melanogaster

**Figure 1:**
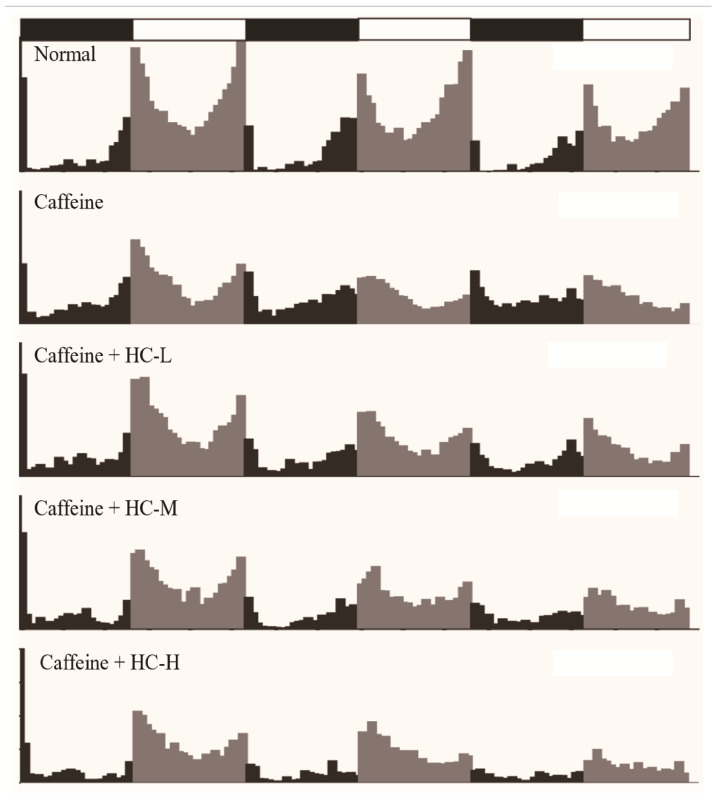
The effect of HC on the overview of locomotor activities in *D. melanogaster* during the 3-day intervention period. Black represents night activity, and gray represents daytime activity. Normal: agar-sucrose food; Caffeine: agar-sucrose food containing 0.1% caffeine; Caffeine + HC-L: agar-sucrose food containing 0.1% caffeine + 0.625% HC extract; Caffeine + HC-M: agar-sucrose food containing 0.1% caffeine + 1.250% HC extract; Caffeine + HC-H: agar-sucrose food containing 0.1% caffeine + 2.500% HC extract.

**Figure 2:**
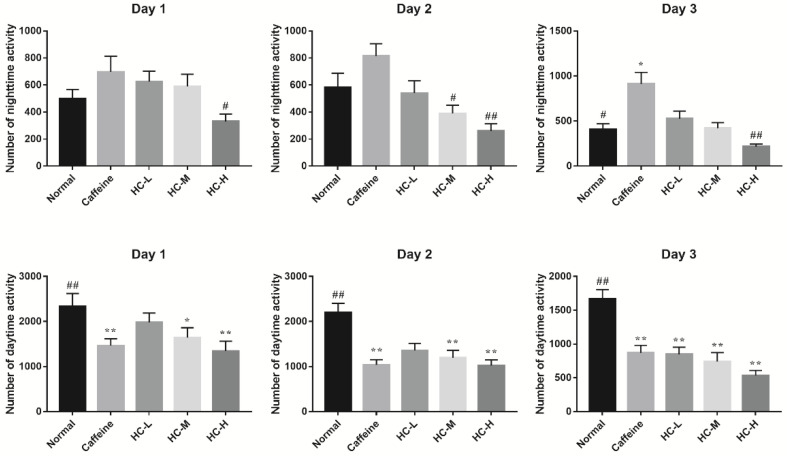
The effect of HC on locomotor activities in D. melanogaster. Caffeine: 1.0 mg/mL of media; HC-L: 0.625% HC extract; HC-M: 1.250% HC extract; HC-H: 2.500% HC extract. * *p* < 0.05 and ** *p* < 0.01 than in the normal group; # *p* < 0.05 and ## *p* < 0.01 than in the caffeine group. Values are mean ± standard error of the mean (SEM).

Fruit flies, like humans, have periodic circadian rhythms and are a well-studied model organism for studying sleep. Meanwhile, caffeine has the same effect on reducing sleep duration in flies as it does in humans [19]. Therefore, in this study, the caffeine-induced insomnia model was used to investigate the effect of HC on locomotor activity in *D. melanogaster* by using the infrared beam-based Drosophila activity monitor (DAM) system. Figure 1 provides an overview of the locomotor activities of each group of *D. melanogaster* over a 3-day period. To investigate the effects of HC on locomotor activity of *D. melanogaster*, the number of nighttime activity and the number of daytime activity was further analyzed (Figure 2). The results revealed that a decreased tendency of nighttime activity was found in a different dose of HC. Among them, HC-H significantly decreased in the nighttime activity of *D. melanogaster* (compared to the caffeine group, *p* < 0.05) on day 1. On day 2, all HC groups significantly decreased in the nighttime activity of *D. melanogaster* (compared to the caffeine group, *p* < 0.05). A similar tendency of nighttime activity was found on day 3. For the amount of daytime activity, compared to the normal group, almost all groups (except the HC-L group on day 1) decreased in the amount of daytime activities from day 1 to day 3 (*p* < 0.05). However, no significant difference was found in all HC groups in comparison with the caffeine group. These results suggest that HC could effectively decrease the nighttime activity of *D. melanogaster* in a dose-dependent manner, while the daytime activity of *D. melanogaster* was not significantly altered when caffeine was administered.

### 3.2. Effect of HC on Sleep Behaviour of D. melanogaster

**Figure 3:**
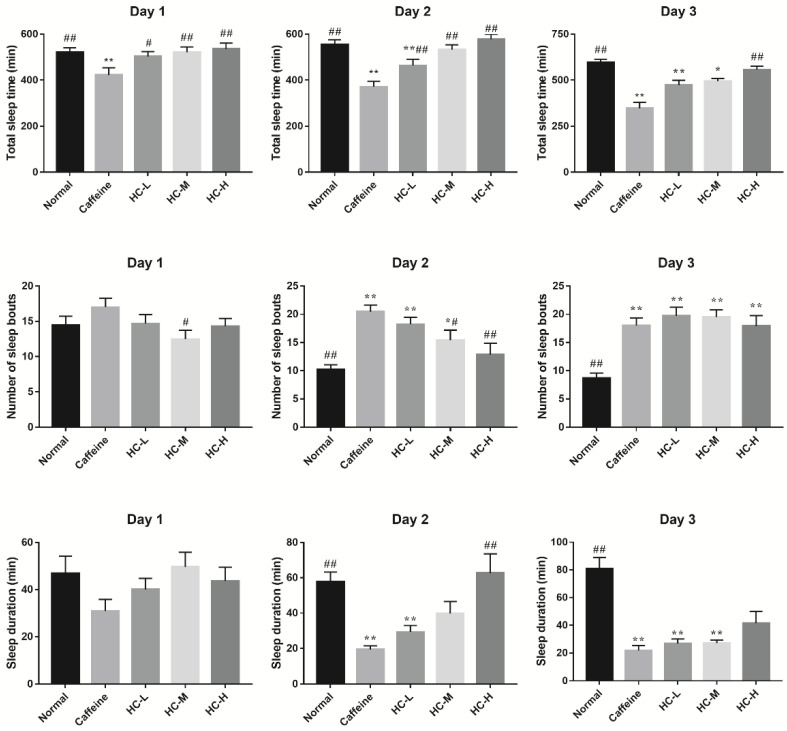
The effect of HC on the sleep-wake behavior of *D. melanogaster*. Caffeine: 1.0 mg/mL of media; HC-L: 0.625% HC extract; HC-M: 1.250% HC extract; HC-H: 2.500% HC extract. * *p* < 0.05 and ** *p* < 0.01 than in the normal group; # *p* < 0.05 and ## *p* < 0.01 than in the caffeine group. Values are mean ± SEM.

For the total sleep time, compared with the normal group, caffeine effectively reduced the total sleep time of *D. melanogaster*, indicating the insomnia model was successfully built (Figure 3). In addition, we found that HC elevated the total sleep time of *D. melanogaster* in a dose-dependent manner over a 3-day period. In particular, all HC groups significantly improve the total sleep time of *D. melanogaster* in comparison with the caffeine group (day 1–day 3, *p* < 0.05), indicating that HC could prolong the sleep time of *D. melanogaster* induced by caffeine.

The number of sleep bouts and sleep duration of *D. melanogaster* during the night can provide some indication of sleep quality [13]. Compared with the caffeine group, all HC groups reduced the number of sleep bouts, while only that of the HC-M group was obviously decreased on day 1 (Figure 3). Meanwhile, we found that all HC groups elevated the sleep duration more than that of the caffeine group (Figure 3). Of them, there was a statistical difference between the HC-M group and the caffeine group (*p* < 0.05). On day 2, caffeine elevated the sleep bouts of *D. melanogaster* (*p* < 0.05), while HC reduced the number of sleep bouts in a dose-dependent manner. Among them, the HC-M and HC-H groups significantly reducing the number of sleep bouts compared with the caffeine group (*p* < 0.05). The results of sleep duration provided further evidence that HC improved the sleep duration in a dose-dependent manner (Figure 3). Interestingly, there were no significant changes in the number of sleep bouts among the caffeine group and the HC groups on day 3. In addition, consistent results of sleep duration were found among the caffeine group and the HC groups on day 3. Although there was no significant difference in sleep duration found between the HC-H group and the caffeine group, the average sleep duration of the HC-H group showed an increased tendency. These results indicate that HC could effectively improve total sleep time in a dose-dependent manner and regulate the sleep bouts and sleep duration in caffeine-induced insomnia in *D. melanogaster*.

### 3.3. UHPLC-ESI-Orbitrap-MS Analysis of HC and Compound Identification

To identify the compounds of HC, the optimized LC-MS conditions were adopted for the characterization of HC chemical compounds. Furthermore, both positive ESI modes and negative ESI modes were used to identify compounds of HC as comprehensively as possible (Figures S1 and S2). By importing the data into Tracefinder 3.4, loaded with the OTCML database, the components of HC were identified. Those components owned identical *m*/*z*, fragment ions, and isotopes to the reference compounds. As a result, 57 compounds of HC were identified from the OTCML database, mainly including flavonoids (28), phenolic acids (10), nucleotides (8), amino acids (5), and coumarins (3). The specific matching information is summarized in Table 1. In previous studies, Sun et al. preliminary identified 27 phenolic compounds of flowers of daylily (*Hemerocallis fulva* (L.)), indicating that rutin, kaempferol-3-O-rutinoside, 5-O-caffeoylquinic acid, and 5-O-p-coumaroylquinic acid were its major phenolic compounds [39]. The present study performed a comprehensive phytochemical scan and identified more types of plant compounds, not limited to polyphenols, which laid a firm basis for the subsequent network pharmacology analysis.

### 3.4. Screening of the Candidate Targets

**Figure 4:**
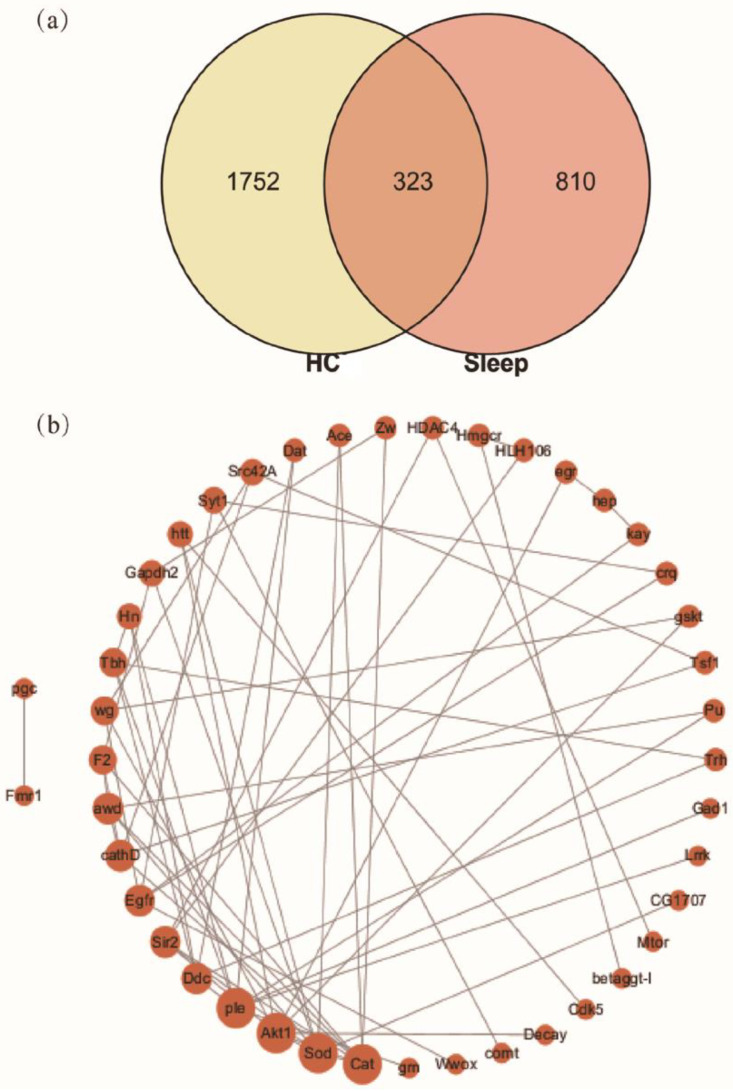
(**a**) The intersection targets of HC and sleep; (**b**) the protein-protein interaction (PPI) network of D. melanogaster constructed by intersection targets. The size of nodes represents the value of degree.

As a complex system with multiple components, HC may play a role in promoting health through multiple targets and multiple pathways. Network pharmacology is a new strategy based on system pharmacology and bioinformatics [57], and thus it was applied to screen the candidate sleep-improvement compounds of HC. For the collection of potential targets for HC, a total of 2075 candidate targets (Homo sapiens) from the 56 active components of HC were selected by applying the database for TCMSP, Swiss Target Prediction, SymMap, and PubChem, respectively (Table S2). For the collection of therapeutic targets of sleep-related indications, 1132 targets associated with sleep-related indications were screened from the database for DrugBank, DisGeNET, and Genecards, respectively (Table S3). Eventually, a Venn diagram was constructed to visualize the intersection between compound targets and sleep-related targets. As a result, 323 overlapping targets were screened (Figure 4, Tables S4 and S6), indicating 323 candidate targets of HC compounds were hit from 1132 known therapeutic targets of sleep-related indications.

### 3.5. Functional Enrichment of GO Terms and KEGG Pathway

**Figure 5:**
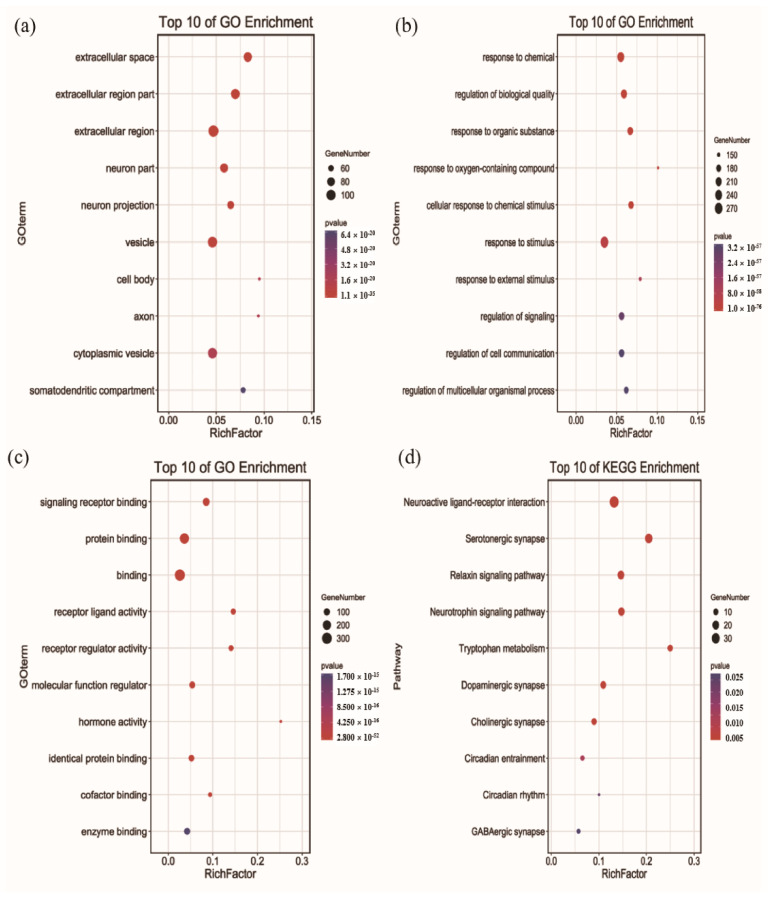
Functional enrichment analysis for GO terms and KEGG pathways (Homo sapiens): (**a**) Cellular Component, (**b**) Biological Process, (**c**) Molecular Function, (**d**) KEGG pathways.

To unveil the sleep-improvement mechanisms of HC, the top 10 GO terms for CC, BP, and MF were picked out based on the *p*-value (Figure 5). For CC, the extracellular space, extracellular region part, extracellular region, neuron part, neuron projection, vesicle, cell body, axon, cytoplasmic vesicle, and somatodendritic compartment were significantly enriched (*p* < 0.05). Furthermore, the results of GO terms showed that overlapping targets were enriched in the BP, including response to chemicals, regulation of biological quality, response to organic substances, response to oxygen-containing compounds, cellular response to chemical stimulus, response to stimulus, response to external stimulus, regulation of signaling, regulation of cell communication, and regulation of multicellular organismal processes (*p* < 0.05). For MF, signaling receptor binding, protein binding, binding, receptor ligand activity, receptor regulator activity, molecular function regulator, hormone activity, identical protein binding, cofactor binding, and enzyme binding were significantly enriched (*p* < 0.05).

To gain further insight into the relationship between sleep and HC, KEGG pathway enrichment analysis was carried out. As a result, 10 remarkable pathways related to sleep were selected, which mainly involved the neuroactive ligand-receptor interaction, serotonergic synapse, relaxin signaling pathway, neurotrophin signaling pathway, tryptophan metabolism, dopaminergic synapse, cholinergic synapse, circadian entrainment, circadian rhythm, and GABAergic synapse (*p* < 0.05). Among them, neuroactive ligand-receptor interaction was the most important topological pathway, and is closely associated with neurological function [58]. Furthermore, the neurotransmitters for serotonin, GABA, acetylcholine, and dopamine play key functions in regulating sleep and wakefulness [59,60,61]. These results further reveal that the sleep-improvement effects of HC involve multiple targets and multiple pathways.

### 3.6. Construction of the C-T-P Network

To uncover the interactions among identified compounds, overlapping targets, and signaling pathways, a C-T-P network was constructed (Figure S3). The results showed that the C-T-P network consists of 387 nodes and 1747 edges, indicating HC possesses the characteristics of multi-compounds, multi-targets, and multi-pathways for sleep-improvement. In particular, three important topological properties of “Degree”, “Betweenness centrality”, and “Closeness centrality” were further analyzed to uncover the important compounds and targets of HC in sleep-improvement (Table 2 and Table S5). For the target, the mean degree value of all candidate targets was 5.41, and the network analysis showed that Tnf (degree = 46), Akt1 (degree = 39), ins (degree = 38), il6 (degree = 37), cat (degree = 36), alb (degree = 35), sod1 (degree = 35), ache (degree = 33), ptgs2 (degree = 33), and tp53 (degree = 33) were the top 10 targets in the C-T-P network, indicating these targets of HC may be the key targets in sleep-improvement. For the compound, the mean degree value of all identified compounds was 29.70, and the quercetin (degree = 127), luteolin (degree = 70), kaempferol (degree = 59), caffeic acid (degree = 59), nicotinic acid (degree = 56), myricetin (degree = 55), chlorogenic acid (degree = 53), s-(-)-carbidopa (degree = 50), rutin (degree = 47), and hyperoside (degree = 45) were the top 10 identified compounds in terms of degree, and they may be the key compounds of HC in sleep-improvement because of their crucial positions in the C-T-P network. Previous studies have reported that quercetin and rutin can alter the sleep-wake cycle by activating GABA(A) receptors [29,62], and luteolin has hypnotic efficacy through ADORA1 and ADORA2A binding [63]. Both caffeic acid and chlorogenic acid significantly prolong sleep latency in rats [33], and nicotinic acid promotes sleep through prostaglandin synthesis [64]. Furthermore, myricetin modulates the GABA(A) receptor activity [65], and hyperoside shows an inhibitory effect on the central nervous system by mediating the dopaminergic system [30]. All of these findings provide further evidence for our results that quercetin, luteolin, kaempferol, caffeic acid, nicotinic acid, myricetin, chlorogenic acid, s-(-)-carbidopa, rutin, and hyperoside are the main active compounds of HC in sleep-improvement.

### 3.7. Effects of HC on mRNA Levels in D. melanogaster

**Figure 6:**
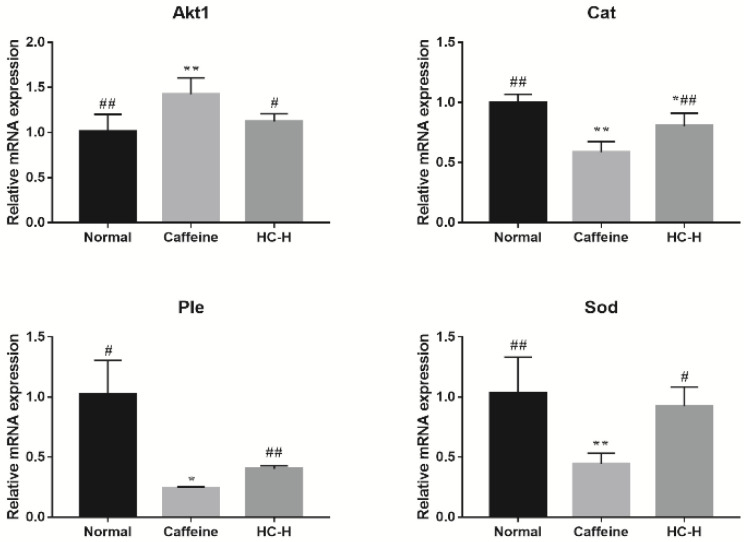
The effect of HC on core target mRNA expression in the heads of *D. melanogaster*. Caffeine: 1.0 mg/mL of media; HC-H: 2.500% HC extract. * *p* < 0.05 and ** *p* < 0.01 than in the normal group; # *p* < 0.05 and ## *p* < 0.01 than in the caffeine group. Values are mean ± standard deviation (SD).

To further validate the core targets of the Drosophila-associated PPI networks affected by HC, the top 4-degree targets (Akt1, Sod, Cat, and Ple) were selected (Figure 4b). In this study, the transcript levels for the Akt1 in the caffeine group were significantly higher than for the normal group (Figure 6). However, HC treatment significantly reduced the level of Akt1. The previous study reported that Akt pathways are a major regulator of nutrient metabolism, cell growth, and senescence, and they impact the brain circadian clock that drives behavioral rhythms in *D. melanogaster* [66] and the rhythmic expression of Akt1 in the skeletal muscle of mice [67]. These results suggest that HC can ameliorate the changes in Akt1 transcription levels induced by caffeine. In addition, the *D. melanogaster* exposed to caffeine had significantly decreased transcript levels for Cat, Ple, and Sod (Figure 6), while the administration of HC effectively reversed this trend (*p* < 0.05). The previous studies have reported the effects of some flavonoids on the temporal regulation of redox homeostasis [68,69]. Among them, hesperidin increased the levels of the antioxidant index (Sod and Cat) and regulated their amplitudes in the rotenone-induced oxidative stress model of Drosophila [69]. Furthermore, the synthesis of dopamine was regulated by expression of Ple [70], and dopamine has several roles in the modulation of locomotor behaviors and arousal states [71,72]. Therefore, HC positively regulated the expression level of Sod, Cat, and Ple in the head of *D. melanogaster* to play a role in sleep-improvement.

## 4. Conclusions

In this work, the results suggested that HC extract effectively regulated the number of nighttime activities and total sleep time of *D. melanogaster* in a dose-dependent manner and positively regulated the sleep bouts and sleep duration of *D. melanogaster*. In addition, the chemical constituents of HC were full-scale identified by UHPLC-ESI-Orbitrap, and 57 compounds were identified. Among them, C-T-P network topology analysis revealed that quercetin, luteolin, kaempferol, caffeic acid, nicotinic acid, myricetin, chlorogenic acid, s-(-)-carbidopa, and rutin were the key compounds of HC in sleep-improvement. Moreover, the core targets (Akt1, Sod, Cat, and Ple) of the Drosophila-associated PPI networks affected by HC were verified by transcriptional levels. In a word, this study revealed that HC plays a beneficial role in sleep improvement through multiple compounds, multiple targets, and multiple pathways, which provides a new perspective on the active components and molecular mechanism of HC. 

## Figures and Tables

**Table 1:** Identification of the chemical components of *Hemerocallis citrina* Baroni by UHPLC-ESI-Orbitrap-MS.

Compound Name	RT (min)	Molecular Formula	Measured Mass (*m*/*z*)	ESI-MS	Accuracy (ppm)	MS/MS (*m*/*z*)	Category
5-Hydroxymethylfurfural	1.61	C_6_H_6_O_3_	127.04	[M + H]^+^	−2.21	109.03, 81.03	Others
2-Pyrrolidinecarboxylic acid	1.64	C_5_H_9_NO_2_	116.07	[M + H]^+^	−2.43	70.07	Amino acids
Cytosine	1.64	C_4_H_5_N_3_O	112.05	[M + H]^+^	−2.48	95.02, 69.04	Nucleotides
Uridine	2.25	C_9_H_12_N_2_O_6_	243.06	[M − H]^−^	−2.15	200.06, 152.03	Nucleotides
Adenosine	2.25	C_10_H_13_N_5_O_4_	268.1	[M + H]^+^	−2.72	136.06	Nucleotides
Guanine	2.25	C_5_H_5_N_5_O	152.06	[M + H]^+^	−1.83	135.03, 110.03	Nucleotides
Guanosine	2.25	C_10_H_13_N_5_O_5_	284.1	[M + H]^+^	−3.16	152.06	Nucleotides
L-Tyrosine	2.25	C_9_H_11_NO_3_	182.08	[M + H]^+^	−1.72	136.08, 123.04	Amino acids
Cordycepin	2.25	C_10_H_13_N_5_O_3_	252.11	[M + H]^+^	−2.5	136.06, 99.04	Nucleotides
Nicotinic acid	2.25	C_6_H_5_NO_2_	124.04	[M + H]^+^	−1.91	96.04, 80.05	Vitamin
Isoguanosine	2.27	C_10_H_13_N_5_O_5_	282.08	[M − H]^-^	−0.90	150.04, 107.03	Nucleotides
L-Leucine	2.8	C_6_H_13_NO_2_	132.1	[M + H]^+^	−1.59	86.1, 69.07	Amino acids
S-(-)-Carbidopa	2.83	C_10_H_14_N_2_O_4_	225.09	[M − H]^−^	−2.67	164.07	Phenolic acids
Thymidine	3.25	C_10_H_14_N_2_O_5_	241.08	[M − H]^−^	−1.26	151.05, 125.03	Nucleotides
L-Phenylalanine	3.95	C_9_H_11_NO_2_	166.09	[M + H]^+^	−1.21	149.06, 120.08	Amino acids
7-Hydroxycoumarin	5.38	C_9_H_6_O_3_	163.04	[M + H]^+^	−1.21	107.05	Coumarins
Neochlorogenic acid	5.38	C_16_H_18_O_9_	355.1	[M + H]^+^	−2.07	163.04	Phenolic acids
5-Acetylsalicylic acid	5.4	C_9_H_8_O_4_	181.05	[M + H]^+^	−1.57	107.05	Phenolic acids
Caffeic acid	5.4	C_9_H_8_O_4_	181.05	[M + H]^+^	−1.57	135.04, 117.03	Phenolic acids
1-Caffeoylquinic acid	5.47	C_16_H_18_O_9_	353.09	[M − H]^−^	−0.55	191.06, 179.03	Phenolic acids
Chlorogenic acid	5.47	C_16_H_18_O_9_	353.09	[M − H]^−^	−0.55	191.06, 161.02	Phenolic acids
Cryptochlorogenic acid	5.47	C_16_H_18_O_9_	353.09	[M − H]^−^	−0.55	173.04, 135.04	Phenolic acids
Danshensu	6.24	C_9_H_10_O_5_	197.04	[M − H]^−^	−3.96	179.03, 135.04	Phenolic acids
L-Tryptophan	6.52	C_11_H_12_N_2_O_2_	205.1	[M + H]^+^	−1.33	146.06, 118.06	Amino acids
4-Methylumbelliferone	8.68	C_10_H_8_O_3_	177.05	[M + H]^+^	−0.65	149.06	Coumarins
7-Methoxycoumarin	8.68	C_10_H_8_O_3_	177.05	[M + H]^+^	−0.65	149.06, 121.06	Coumarins
Androsin	10.39	C_15_H_20_O_8_	327.11	[M − H]^−^	0.50	165.05	Others
Grosvenorine	14.17	C_33_H_40_O_19_	741.22	[M + H]^+^	−1.60	287.05	Flavonoids
Typhaneoside	14.53	C_34_H_42_O_20_	769.22	[M − H]^−^	0.95	314.04, 151.00	Flavonoids
Rutin	14.63	C_27_H_30_O_16_	609.15	[M − H]^−^	0.31	300.03	Flavonoids
Taxifolin	14.81	C_15_H_12_O_7_	303.05	[M − H]^−^	0.29	241.05	Flavonoids
Hyperoside	15.27	C_21_H_20_O_12_	463.09	[M − H]^−^	0.72	300.03, 271.02	Flavonoids
Isoquercitrin	15.27	C_21_H_20_O_12_	463.09	[M − H]^−^	0.72	300.03, 271.02	Flavonoids
Myricitrin	15.27	C_21_H_20_O_12_	463.09	[M − H]^−^	0.72	271.02	Flavonoids
5-Hydroxy-1-tetralone	16.72	C_10_H_10_O_2_	163.08	[M + H]^+^	−1.76	135.08, 107.05	Flavonoids
Oroxin B	16.76	C_27_H_30_O_15_	595.16	[M + H]^+^	−1.50	145.05	Flavonoids
Kaempferol	16.76	C_15_H_10_O_6_	287.05	[M + H]^+^	−2.40	153.02	Flavonoids
Luteolin	16.76	C_15_H_10_O_6_	287.05	[M + H]^+^	−2.40	153.02	Flavonoids
Kaempferol 3-glucorhamnoside	16.77	C_27_H_30_O_15_	593.15	[M − H]^−^	−0.42	327.05, 284.03	Flavonoids
Kaempferol-3-O-rutinoside	16.77	C_27_H_30_O_15_	593.15	[M − H]^−^	−0.42	285.04, 255.03	Flavonoids
Lonicerin	16.77	C_27_H_30_O_15_	593.15	[M − H]^−^	−0.42	285.04	Flavonoids
Isorhamnetin-3-O-nehesperidine	17.32	C_28_H_32_O_16_	623.16	[M − H]^−^	1.25	314.04, 271.03	Flavonoids
Narcissoside	17.32	C_28_H_32_O_16_	623.16	[M − H]^−^	1.25	315.05, 271.03	Flavonoids
Astragalin	17.45	C_21_H_20_O_11_	447.09	[M − H]^−^	0.30	284.03, 227.03	Flavonoids
Cynaroside	17.45	C_21_H_20_O_11_	447.09	[M − H]^−^	0.30	285.04, 151.00	Flavonoids
Homoorientin	17.45	C_21_H_20_O_11_	447.09	[M − H]^−^	0.30	327.05	Flavonoids
Kaempferol-7-O-β-D-glucopyranoside	17.45	C_21_H_20_O_11_	447.09	[M − H]^−^	0.30	285.04, 151.00	Flavonoids
Orientin	17.45	C_21_H_20_O_11_	447.09	[M − H]^−^	0.30	327.05	Flavonoids
Quercetin 7-rhamnoside	17.45	C_21_H_20_O_11_	447.09	[M − H]^−^	0.30	301.04	Flavonoids
Quercitrin	17.45	C_21_H_20_O_11_	447.09	[M − H]^−^	0.30	300.03	Flavonoids
Eriodictyol	18.28	C_15_H_12_O_6_	287.06	[M − H]^−^	0.57	151	Flavonoids
Dehydrodiisoeugenol	19.6	C_20_H_22_O_4_	327.16	[M + H]^+^	−2.52	203.11	Phenolic acids
Nepodin	20.49	C_13_H_12_O_3_	217.09	[M + H]^+^	−0.55	184.05, 171.08	Phenolic acids
Morin	21.21	C_15_H_10_O_7_	301.04	[M − H]^−^	−0.03	151, 107.01	Flavonoids
Quercetin	21.21	C_15_H_10_O_7_	301.04	[M − H]^−^	−0.03	179.00, 151.00	Flavonoids
Demethylwedelolactone	21.21	C_15_H_8_O_7_	299.02	[M − H]^−^	0.25	271.02	Flavonoids
Myricetin	21.21	C_15_H_10_O_8_	317.03	[M − H]^−^	−0.44	179.00, 151.00	Flavonoids

RT: retention time.

**Table 2:** The C-T-P network topology analysis for top 20 compounds.

Compound	Degree	Betweenness Centrality	Closeness Centrality
Quercetin	127	0.26	0.46
Luteolin	70	0.06	0.40
Kaempferol	59	0.04	0.39
Caffeic acid	59	0.06	0.39
Nicotinic acid	56	0.07	0.39
Myricetin	55	0.03	0.39
Chlorogenic acid	53	0.04	0.39
S-(-)-Carbidopa	50	0.09	0.38
Rutin	47	0.03	0.38
Hyperoside	45	0.03	0.38
Adenosine	45	0.06	0.38
Myricitrin	43	0.06	0.38
Danshensu	42	0.03	0.38
Isoquercitrin	40	0.01	0.38
Thymidine	39	0.03	0.38
Cordycepin	39	0.03	0.38
L-Tryptophan	36	0.04	0.37
7-Hydroxycoumarin	35	0.01	0.38
Kaempferol-3-O-rutinoside	33	0.02	0.37
Cynaroside	33	0.01	0.37

## Data Availability

Data is contained within the article or supplementary material.

## Supplementary Materials

The following are available online at https://www.mdpi.com/article/10.3390/foods10040883/s1, Figure S1: Base peak chromatograms of compounds from HC by UPLC-Orbitrap-MS in ESI−, Figure S2: Base peak chromatograms of compounds from HC by UPLC-Orbitrap-MS in ESI+, Figure S3: The Component-Target-Pathway (C-T-P) network (*Homo sapiens*). Blue hexagons represent top 10 pathways, green diamonds represent components of HC, and red rounds represent overlapped targets, Table S1: RT-PCR primer design sequence, Table S2: Candidate targets for HC, Table S3: Therapeutic targets for sleep related indications, Table S4: Intersection between targets of HC and targets of sleep, Table S5: The C-T-P network topology analysis for top 20 targets, Table S6: The PPI network of *D. melanogaster* constructed by intersection targets.

## References

[1] Saper C.B., Fuller P.M., Pedersen N.P., Lu J., Scammell T.E. (2010). Sleep state switching. Neuron.

[2] Liu W.-L., Wu B.-F., Shang J.-H., Zhao Y.-L., Huang A.-X. (2020). Lam seed oil augments pentobarbital-induced sleeping behaviors in mice via GABAergic systems. J. Agric. Food Chem..

[3] Kwon Y.O., Hong J.T., Oh K.-W. (2017). Rosmarinic acid potentiates pentobarbital-induced sleep behaviors and Non-Rapid Eye Movement (NREM) sleep through the activation of GABA-ergic systems. Biomol. Ther..

[4] Fernandez-Mendoza J., Shea S., Vgontzas A.N., Calhoun S.L., Liao D., Bixler E.O. (2015). Insomnia and incident depression: Role of objective sleep duration and natural history. J. Sleep Res..

[5] Sivertsen B., Lallukka T., Salo P., Pallesen S., Hysing M., Krokstad S., Simon Ø. (2014). Insomnia as a risk factor for ill health: Results from the large population-based prospective HUNT Study in Norway. J. Sleep Res..

[6] Blank M., Zhang J., Lamers F., Taylor A.D., Hickie I.B., Merikangas K.R. (2015). Health correlates of insomnia symptoms and comorbid mental disorders in a nationally representative sample of US adolescents. Sleep.

[7] Zhuang J., Zhan Y., Zhang F., Tang Z., Wang J., Sun Y., Ding R., Hu D., Yu J. (2016). Self-reported insomnia and coronary heart disease in the elderly. Clin. Exp. Hypertens..

[8] Kay-Stacey M., Attarian H. (2016). Advances in the management of chronic insomnia. BMJ.

[9] Chung K.-F., Yeung W.-F., Ho F.Y.-Y., Yung K.-P., Yu Y.-M., Kwok C.-W. (2015). Cross-cultural and comparative epidemiology of insomnia: The Diagnostic and statistical manual (DSM), International classification of diseases (ICD) and International classification of sleep disorders (ICSD). Sleep Med..

[10] Ford E.S., Cunningham T.J., Giles W.H., Croft J.B. (2015). Trends in insomnia and excessive daytime sleepiness among U.S. adults from 2002 to 2012. Sleep Med..

[11] Léger D., Partinen M., Hirshkowitz M., Chokroverty S., Hedner J. (2010). Characteristics of insomnia in a primary care setting: EQUINOX survey of 5293 insomniacs from 10 countries. Sleep Med..

[12] Weaver M.F. (2015). Prescription sedative misuse and abuse. Yale J. Biol. Med..

[13] Ki Y., Lim C. (2019). Sleep-promoting effects of threonine link amino acid metabolism in *Drosophila* neuron to GABAergic control of sleep drive. Elife.

[14] Guadagna S., Barattini D.F., Rosu S., Ferini-Strambi L. (2020). Plant extracts for sleep disturbances: A systematic review. Evid. Based Complement. Altern. Med..

[15] Kim J., Lee S.L., Kang I., Song Y.A., Ma J., Hong Y.S., Park S., Moon S.I., Kim S., Jeong S. (2018). Natural products from single plants as sleep aids: A systematic review. J. Med. Food.

[16] Hong K.B., Park Y., Suh H.J. (2016). Sleep-promoting effects of a GABA/5-HTP mixture: Behavioral changes and neuromodulation in an invertebrate model. Life Sci..

[17] Dubowy C., Sehgal A. (2017). Circadian rhythms and sleep in *Drosophila melanogaster*. Genetics.

[18] Staats S., Luersen K., Wagner A.E., Rimbach G. (2018). *Drosophila melanogaster* as a versatile model organism in food and nutrition research. J. Agric. Food Chem..

[19] Ko B.S., Ahn S.H., Noh D.O., Hong K.B., Han S.H., Suh H.J. (2017). Effect of explosion-puffed coffee on locomotor activity and behavioral patterns in *Drosophila melanogaster*. Food Res. Int..

[20] Zhang Z.-Q., Degejin, Geng D., Zhang Q., Tian Y., Xi Y., Wang W.-Q., Tang H.-Q., Xu B., Lin H.-Y. (2016). Pharmacodynamic study on insomnia-curing effects of Shuangxia Decoction in *Drosophila melanogaster*. Chin. J. Nat. Med..

[21] Matraszek-Gawron R., Chwil M., Terlecka P., Skoczylas M.M. (2019). Recent studies on anti-depressant bioactive substances in selected species from the genera *Hemerocallis* and *Gladiolus*: A systematic review. Pharmaceuticals.

[22] Du B., Tang X., Liu F., Zhang C., Zhao G., Ren F., Leng X. (2014). Antidepressant-like effects of the hydroalcoholic extracts of *Hemerocallis citrina* and its potential active components. BMC Complement. Altern. Med..

[23] Xu P., Wang K.Z., Lu C., Dong L.M., Zhai J.L., Liao Y.H., Aibai S., Yang Y.Y., Liu X.M. (2016). Antidepressant-like effects and cognitive enhancement of the total phenols extract of *Hemerocallis citrina* Baroni in chronic unpredictable mild stress rats and its related mechanism. J. Ethnopharmacol..

[24] Liu J.H., Zhong X.H., Jiang Y.Y., Yu L.Y., Huang X.Q., Dong Z., Yang S.Y., He W., Zeng J.G., Qing Z.X. (2020). Systematic identification metabolites of *Hemerocallis citrina* Borani by high-performance liquid chromatography/quadrupole-time-of-flight mass spectrometry combined with a screening method. J. Pharm. Biomed. Anal..

[25] Li C.F., Chen X.Q., Chen S.M., Chen X.M., Di G., Liu Q., Yi L.T. (2017). Evaluation of the toxicological properties and anti-inflammatory mechanism of *Hemerocallis citrina* in LPS-induced depressive-like mice. Biomed. Pharmacother..

[26] Liu X.L., Luo L., Liu B.B., Li J., Geng D., Liu Q., Yi L.T. (2014). Ethanol extracts from *Hemerocallis citrina* attenuate the upregulation of proinflammatory cytokines and indoleamine 2,3-dioxygenase in rats. J. Ethnopharmacol..

[27] Gu L., Liu Y.J., Wang Y.B., Yi L.T. (2012). Role for monoaminergic systems in the antidepressant-like effect of ethanol extracts from *Hemerocallis citrina*. J. Ethnopharmacol..

[28] Li C.F., Chen S.M., Chen X.M., Mu R.H., Wang S.S., Geng D., Liu Q., Yi L.T. (2016). ERK-dependent brain-derived neurotrophic factor regulation by hesperidin in mice exposed to chronic mild stress. Brain Res. Bull..

[29] Can O.D., Ozkay U.D. (2012). Effects of *Hypericum montbretti* extract on the central nervous system and involvement of GABA (A)/Benzodiazepine receptors in its pharmacological activity. Phytother. Res..

[30] Haas J.S., Stolz E.D., Betti A.H., Stein A.C., Schripsema J., von Poser G.L., Rates S.M.K. (2011). The Anti-immobility effect of hyperoside on the forced swimming test in rats is mediated by the D2-Like receptors activation. Planta Med..

[31] Alonso-Castro A.J., Gasca-Martínez D., Cortez-Mendoza L.V., Alba-Betancourt C., Ruiz-Padilla A.J., Zapata-Morales J.R. (2020). Evaluation of the neuropharmacological effects of Gardenin A in mice. Drug Dev. Res..

[32] Wang W.J., Yang L.D., Liu T.L., Wang J.W., Wen A.D., Ding Y. (2020). Ellagic acid protects mice against sleep deprivation-induced memory impairment and anxiety by inhibiting TLR4 and activating Nrf2. Aging-US.

[33] Shinomiya K., Omichi J., Ohnishi R., Ito H., Yoshida T., Kamei C. (2004). Effects of chlorogenic acid and its metabolites on the sleep-wakefulness cycle in rats. Eur. J. Pharmacol..

[34] Hopkins A.L. (2008). Network pharmacology: The next paradigm in drug discovery. Nat. Chem. Biol..

[35] Hopkins A.L. (2007). Network pharmacology. Nat. Biotechnol..

[36] Wang L.L., Li Z., Zhao X.P., Liu W., Liu Y.F., Yang J.H., Li X., Fan X.H., Cheng Y.Y. (2013). A network study of Chinese Medicine Xuesaitong Injection to elucidate a complex mode of action with multicompound, multitarget, and multipathway. Evid. Based Complement. Altern. Med..

[37] Andretic R., Shaw P.J., Young M.W. (2005). Essentials of sleep recordings in *Drosophila*: Moving beyond sleep time. Circadian Rhythms.

[38] Ko C.H., Koon C.M., Yu S.L., Lee K.Y., Lau C.B., Chan E.H., Wing Y.K., Fung K.P., Leung P.C. (2016). Hypnotic effects of a novel anti-insomnia formula on *Drosophila* insomnia model. Chin. J. Integr. Med..

[39] Sun J., Liu W., Zhang M., Geng P., Shan Y., Li G., Zhao Y., Chen P. (2018). The analysis of phenolic compounds in daylily using UHPLC-HRMSnand evaluation of drying processing method by fingerprinting and metabolomic approaches. J. Food Process. Preserv..

[40] Barbosa S., Saurina J., Puignou L., Núñez O. (2020). Targeted UHPLC-HRMS (Orbitrap) polyphenolic and capsaicinoid profiling for the chemometric characterization and classification of paprika with protected designation of origin (PDO) attributes. Molecules.

[41] Liang X., Liu C.-S., Xia T., Tang Q.-F., Tan X.-M. (2020). Identification of active compounds of Mahuang Fuzi Xixin Decoction and their mechanisms of action by LC-MS/MS and network pharmacology. Evid. Based Complement. Altern. Med. eCAM.

[42] Ru J., Li P., Wang J., Zhou W., Li B., Huang C., Li P., Guo Z., Tao W., Yang Y. (2014). TCMSP: A database of systems pharmacology for drug discovery from herbal medicines. J. Cheminform..

[43] Daina A., Michielin O., Zoete V. (2019). SwissTargetPrediction: Updated data and new features for efficient prediction of protein targets of small molecules. Nucleic Acids Res..

[44] Wu Y., Zhang F., Yang K., Fang S., Bu D., Li H., Sun L., Hu H., Gao K., Wang W. (2019). SymMap: An integrative database of traditional Chinese medicine enhanced by symptom mapping. Nucleic Acids Res..

[45] Ming H., Tiejun C., Yanli W., Stephen B.H. (2013). Web search and data mining of natural products and their bioactivities in PubChem. Sci. China Chem..

[46] Stelzer G., Rosen N., Plaschkes I., Zimmerman S., Twik M., Fishilevich S., Stein T.I., Nudel R., Lieder I., Mazor Y. (2016). The GeneCards Suite: From gene data mining to disease genome sequence analyses. Curr. Protoc. Bioinform..

[47] Wishart D.S., Feunang Y.D., Guo A.C., Lo E.J., Marcu A., Grant J.R., Sajed T., Johnson D., Li C., Sayeeda Z. (2018). DrugBank 5.0: A major update to the DrugBank database for 2018. Nucleic Acids Res..

[48] Piñero J., Ramírez-Anguita J.M., Saüch-Pitarch J., Ronzano F., Centeno E., Sanz F., Furlong L.I. (2020). The DisGeNET knowledge platform for disease genomics: 2019 update. Nucleic Acids Res..

[49] Piñero J., Bravo À., Queralt-Rosinach N., Gutiérrez-Sacristán A., Deu-Pons J., Centeno E., García-García J., Sanz F., Furlong L.I. (2017). DisGeNET: A comprehensive platform integrating information on human disease-associated genes and variants. Nucleic Acids Res..

[50] Piñero J., Queralt-Rosinach N., Bravo À., Deu-Pons J., Bauer-Mehren A., Baron M., Sanz F., Furlong L.I. (2015). DisGeNET: A discovery platform for the dynamical exploration of human diseases and their genes. Database.

[51] Szklarczyk D., Gable A.L., Lyon D., Junge A., Wyder S., Huerta-Cepas J., Simonovic M., Doncheva N.T., Morris J.H., Bork P. (2019). STRING v11: Protein-protein association networks with increased coverage, supporting functional discovery in genome-wide experimental datasets. Nucleic Acids Res..

[52] Huang P., Ke H., Qiu Y., Cai M., Qu J., Leng A. (2019). systematically characterizing chemical profile and potential mechanisms of Qingre Lidan Decoction acting on cholelithiasis by integrating UHPLC-QTOF-MS and network target analysis. Evid. Based Complement. Altern. Med. eCAM.

[53] Yu G., Wang W., Wang X., Xu M., Zhang L., Ding L., Guo R., Shi Y. (2018). Network pharmacology-based strategy to investigate pharmacological mechanisms of Zuojinwan for treatment of gastritis. BMC Complement. Altern. Med..

[54] Azuaje F.J., Zhang L., Devaux Y., Wagner D.R. (2011). Drug-target network in myocardial infarction reveals multiple side effects of unrelated drugs. Sci. Rep..

[55] Chen S.Y., Yang Q., Chen X., Tian Y.Q., Liu Z.Y., Wang S.Y. (2020). Bioactive peptides derived from crimson snapper and in vivo anti-aging effects on fat diet-induced high fat *Drosophila melanogaster*. Food Funct..

[56] Zhang J.J., Liu X., Pan J.H., Zhao Q., Li Y.M., Gao W.G., Zhang Z.S. (2020). Anti-aging effect of brown black wolfberry on Drosophila melanogaster and D-galactose-induced aging mice. J. Funct. Foods.

[57] Mao T., Zhang J., Qiao Y., Liu B., Zhang S. (2019). Uncovering synergistic mechanism of Chinese Herbal Medicine in the treatment of atrial fibrillation with obstructive sleep apnea hypopnea syndrome by network pharmacology. Evid. Based Complement. Altern. Med. eCAM.

[58] Adkins D.E., Khachane A.N., McClay J.L., Aberg K., Bukszár J., Sullivan P.F., van den Oord E.J.C.G. (2012). SNP-based analysis of neuroactive ligand-receptor interaction pathways implicates PGE2 as a novel mediator of antipsychotic treatment response: Data from the CATIE study. Schizophr. Res..

[59] Monti J.M. (2013). The neurotransmitters of sleep and wake, a physiological reviews series. Sleep Med. Rev..

[60] Wu C., Huang Y., Lai X., Lai R., Zhao W., Zhang M., Zhao W. (2014). Study on quality components and sleep-promoting effect of GABA Maoyecha tea. J. Funct. Foods.

[61] Jeon S.J., Park H.J., Gao Q., Lee H.E., Park S.J., Hong E., Jang D.S., Shin C.Y., Cheong J.H., Ryu J.H. (2015). Positive effects of beta-amyrin on pentobarbital-induced sleep in mice via GABAergic neurotransmitter system. Behav. Brain Res..

[62] Kambe D., Kotani M., Yoshimoto M., Kaku S., Chaki S., Honda K. (2010). Effects of quercetin on the sleep-wake cycle in rats: Involvement of gamma-aminobutyric acid receptor type A in regulation of rapid eye movement sleep. Brain Res..

[63] Kim T.H., Custodio R.J., Cheong J.H., Kim H.J., Jung Y.S. (2019). Sleep promoting effect of luteolin in mice via Adenosine A1 and A2A receptors. Biomol. Ther..

[64] Szentirmai E., Kapas L. (2019). Nicotinic acid promotes sleep through prostaglandin synthesis in mice. Sci. Rep..

[65] Zhang X.H., Ma Z.G., Rowlands D.K., Gou Y.L., Fok K.L., Wong H.Y., Yu M.K., Tsang L.L., Mu L., Chen L. (2012). Flavonoid myricetin modulates GABA(A) receptor activity through activation of Ca^2+^ channels and CaMK-II pathway. Evid. Based Complement. Altern. Med..

[66] Zheng X.Z., Sehgal A. (2010). AKT and TOR signaling set the pace of the circadian pacemaker. Curr. Biol..

[67] Shavlakadze T., Anwari T., Soffe Z., Cozens G., Mark P.J., Gondro C., Grounds M.D. (2013). Impact of fasting on the rhythmic expression of myogenic and metabolic factors in skeletal muscle of adult mice. Am. J. Physiol. Cell Physiol..

[68] Subramanian P., Kaliyamoorthy K., Jayapalan J.J., Abdul-Rahman P.S., Hashim O.H. (2017). Influence of quercetin in the temporal regulation of redox homeostasis in *Drosophila melanogaster*. J. Insect Sci..

[69] Arumugam M., Jayapalan J.J., Abdul-Rahman P.S., Hashim O.H., Subramanian P. (2018). Effect of hesperidin on the temporal regulation of redox homeostasis in clock mutant (Cry(b)) of *Drosophila melanogaster*. Biol. Rhythm. Res..

[70] Hirsh J., Riemensperger T., Coulom H., Iche M., Coupar J., Birman S. (2010). Roles of dopamine in circadian rhythmicity and extreme light sensitivity of circadian entrainment. Curr. Biol..

[71] Karam C.S., Jones S.K., Javitch J.A. (2020). Come Fly with Me: An overview of dopamine receptors in *Drosophila melanogaster*. Basic Clin. Pharmacol. Toxicol..

[72] Hanna M.E., Bednarova A., Rakshit K., Chaudhuri A., O’Donnell J.M., Krishnan N. (2015). Perturbations in dopamine synthesis lead to discrete physiological effects and impact oxidative stress response in *Drosophila*. J. Insect Physiol..

