# Supplementary material for: Study on the Sleep-Improvement Effects of Hemerocallis citrina Baroni in Drosophila melanogaster and Targeted Screening to Identify Its Active Components and Mechanism
Source: Foods. 2021 Apr 17;10(4):883. doi: 10.3390/foods10040883 (PMC8072781; doi:10.3390/foods10040883)
Supplement: Supplementary file 1 [file foods-10-00883-s001.zip › Supplementary materials/Supplementary materials.docx]

Figure S1 Base peak chromatograms of compounds from HC by UPLC-Orbitrap-MS in ESI-.

Figure S2 Base peak chromatograms of compounds from HC by UPLC-Orbitrap-MS in ESI+.

The mass spectrum conditions were as follows:

The runtime was from 0 to 22 minutes. Mass spectra were acquired in profile mode with a resolution setting of 70,000. Operation parameters were as follows: source voltage, 4 kV; sheath gas (N2), 40 (arbitrary units); auxiliary gas (N2), 15 (arbitrary units); sweep gas, 2 (arbitrary units); and capillary temperature, 320 °C. Default values were used for most other acquisition parameters (FT Automatic gain control (AGC) target: 3,000,000). HC samples were analysed in full scan mode at a resolution of 70, 000. The scan range in Full MS/dd MS2 mode was from 100 m/z to 1200 m/z. The most intense ions detected during full scan MS triggered data-dependent scanning. Data-dependent scanning was carried out without the use of a parent ion list. The resolution of dd-MS2 mode was 17500, and the AGC target was 10000. An isolation width of 100 amu was used and precursors were fragmented by the normalised collision energy (10 V, 20 V, 30 V) and an activation time of 50 ms. The data processing was achieved using the software XCalibur 4.1 (Thermo Fisher Scientific, Waltham, MA, USA).

The liquid chromatography conditions were as follows:

Chromatographic separation was accomplished with an Agilent InfinityLab Poroshell 120, 4.6 mm × 150 mm, 2.7 µm column were used (Agilent InfinityLab, USA). Gradient elution of analytes was carried out with water/0.2% formic acid (solvent A) and acetonitrile (solvent B) at a constant flow rate of 300 mL/min, and the injection volume was 10 uL. A non-linear gradient was applied – 0 min, 95% B; 0–15 min, 80% B; 15–20 min, 60% B; 20–21 min, 30% B; 21–22 min, 95% B. The column was equilibrated for 3 min to initial conditions.


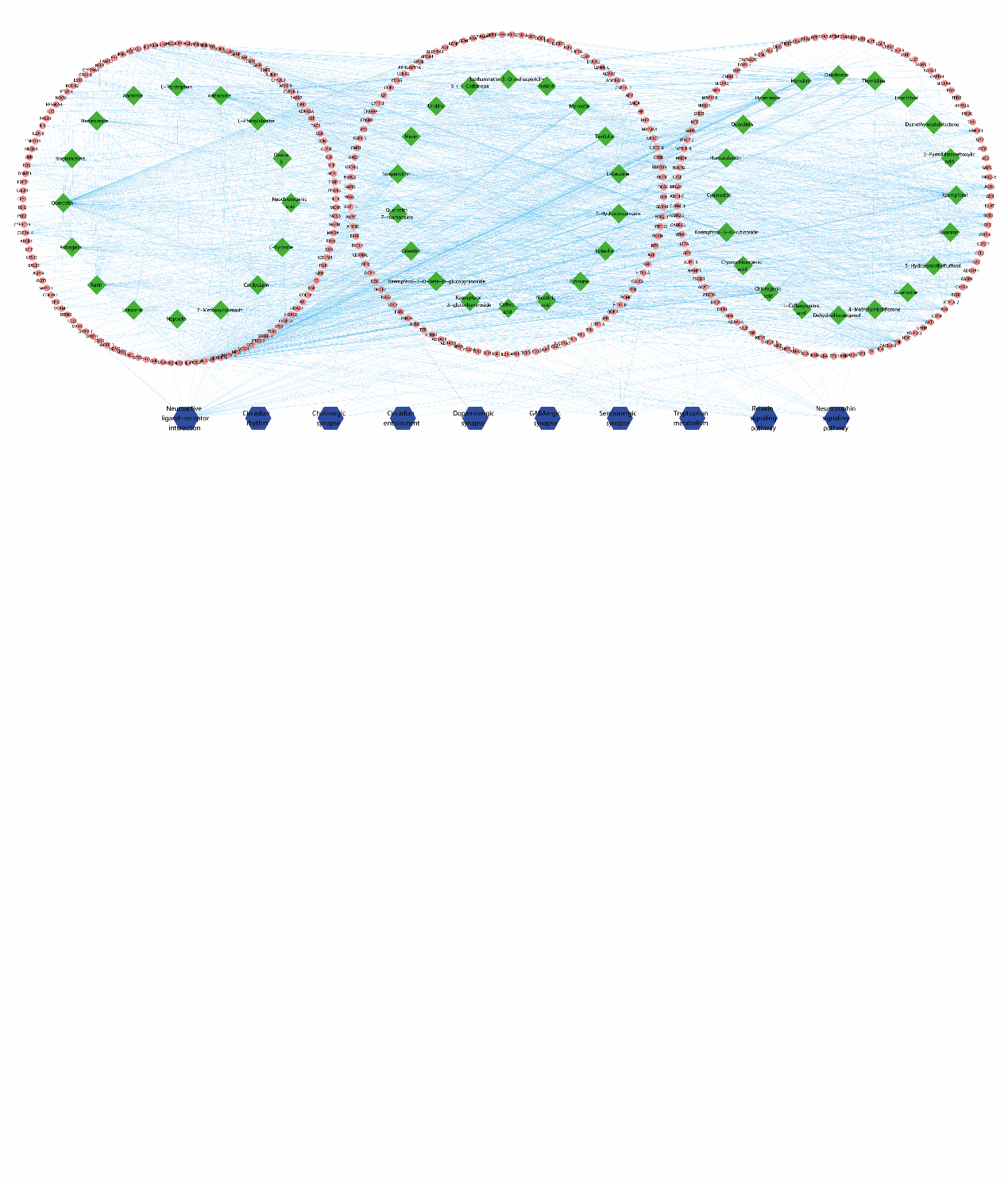


Figure S3 The Component-Target-Pathway (C-T-P) network (Homo sapiens). Blue hexagons represent top 10 pathways, green diamonds represent components of HC and red rounds represent overlapped targets.
